# Resting state connectivity biomarkers of seizure freedom after epilepsy surgery

**DOI:** 10.1016/j.nicl.2024.103673

**Published:** 2024-09-16

**Authors:** Eva Martinez-Lizana, Armin Brandt, Matthias Dümpelmann, Andreas Schulze-Bonhage

**Affiliations:** Epilepsy Center, Medical Center, University of Freiburg, Breisacher Str. 64, 79106 Freiburg im Breisgau, Germany

**Keywords:** Epilepsy, Resting state, Connectivity, Epilepsy surgery, HD-EEG, Source localization, Network measures

## Abstract

Alterations in brain networks may cause the lowering of the seizure threshold and hypersynchronization that underlie the recurrence of unprovoked seizures in epilepsy. The aim of this work is to estimate functional network characteristics, which may help predicting outcome of epilepsy surgery.

Twenty patients were studied (11 females, 9 males, mean age 33 years) with scalp-recorded HD-EEG in resting state (eyes closed, no interictal discharges) before intracranial evaluation, which allowed the precise determination of the epileptogenic zone. Dipole source time courses in the brain were estimated using Weighted Minimum Norm Estimate based on HD-EEG signals. Information inflow and outflow of atlas-based brain regions were computed using partial directed connectivity. A set of graph measures for pairwise connections in standard EEG frequency bands was calculated.

After epilepsy surgery 10 patients were seizure-free (Engel 1a) and 10 patients continued suffering from seizures (Engel outcome worse than 1a). Inflow of the regions containing the epileptogenic zone in the beta and delta frequency bands was significantly lower in patients who achieved seizure-freedom after surgery, compared with patients who continued to have seizures (p = 0.012, and p = 0.026, respectively). Average path length in the beta frequency band was significantly higher in patients who achieved seizure freedom (p = 0.012). In the delta frequency band, local efficiency and clustering coefficient were significantly higher in patients who achieved seizure freedom (0.033, 0.046).

In patients who achieved seizure freedom after surgery, the preoperative analysis of the epileptic network exhibited stronger separation of the region containing the seizure onset zone, with less inflow of information. In contrast, shorter paths within the epileptic network may facilitate hypersynchronous neuronal activity and thus the recurrence of seizures in non-seizure free patients. This study supports the hypothesis that epileptic network properties might help to define suitable candidates for epilepsy surgery.

## Highlights

• Preoperative resting state network analysis in EEG frequency bands may predict epilepsy surgery outcomes.
• Post-surgical seizure freedom correlates with reduced information inflow to the seizure onset zone.
• Structured and clustered epileptic networks predict seizure freedom after epilepsy surgery.
• Extensive and scattered epileptic networks predict poor outcomes after epilepsy surgery.

## 1 Introduction

As the 20th century began, Camillo Golgi and Santiago Ramon y Cajal demonstrated that the nervous system was not an amalgamation of fused cells sharing a common cytoplasm, but rather a highly intricate network of discrete cells possessing the crucial ability to signal to one another. With the advancement of contemporary technology, it is now possible to identify structural brain networks using imaging techniques and functional brain networks using time-series-analysis based on non-invasive and invasive electroencephalograms (EEG), magnetoencephalograms (MEG) or functional magnetic resonance data (Bullmore and Sporns, 2009, Rubinov and Sporns, 2010). Functional magnetic resonance (fMRI) offers an indirect measurement of neuronal activity by assessing oxygen consumption and blood flow. Electroencephalography directly measures neuronal activity at the millisecond timescale, thus offering an excellent temporal resolution to investigate the dynamics of the brain networks. Moreover, EEG-signals in different frequency bands enable the study of different networks and their dynamics.

Research over the last decades has conceptualized epilepsy as a network disorder (Lehnertz et al., 2023, Stam, 2024, Bröhl et al., 2023). Under this new paradigm, epilepsy is rooted in widespread epileptic networks spanning across lobes and hemispheres. This facilitates the comprehension of the epileptic brain, which not only generates seizures but may also cause cognitive and behavioral impairment.

The assessment of structural and functional networks in epileptic patients is used increasingly to understand the neuronal circuit dysfunctions underlying epilepsy. Multiple studies have revealed disease-specific patterns of altered connectivity in the regions where seizures are generated (Coito et al., 2019, von Wrede et al., 2022, Fan et al., 2022). In this context, high density electroencephalography (HD-EEG), with 256 electrodes offers the opportunity to explore the network dynamics with better spatial resolution than conventional EEG (Hassan et al., 2014). Data-driven techniques based on Granger-causality modeling enable the analysis of effective connectivity (Coito et al., 2015).

One third of epilepsy patients do not achieve seizure freedom with anti-seizure medication alone. For these patients, resection of the brain tissue where seizures originate, known as the seizure onset zone (SOZ), is often necessary to get seizures under control. However, in some cases, resection of only the seizure onset zone fails to achieve seizure freedom. This may result from the intricate relationship between the epileptogenic zone and the seizure onset zone, as well as their connections to remote brain areas, which are not fully understood. Recently, electroencephalographic biomarkers of clinical response to epilepsy surgery have been identified using interictal epileptic discharges to analyse connectivity pattern in intracranial recordings (Lagarde et al., 2018, Carboni et al., 2019) or using non-invasive HD-EEG electrical source imaging (Carboni et al., 2019). These biomarkers are derived from interictal epileptic discharges, limiting their clinical utility, as many patients do not have interictal activity detectable in surface EEG. An ideal biomarker would be measurable non-invasively and would not depend on the specific brain region sampled by intracranial electrodes.

Recent studies have demonstrated quantitative analysis techniques for local and global properties of brain networks (Carboni et al., 2019, Taylor et al., 2018, Carboni et al., 2020, Nissen et al., 2016). For a review see the work of Bröhl et al. (Bröhl et al., 2023), van Diessen et al. (Van Diessen et al., 2014) or Bullmore et al (Bullmore et al., 2009b). According to graph theory, the brain is a complex network in which a set of nodes (technically called vertices) represents different brain regions. The edges of this network represent the connections between these regions. From this elementary network various quantitative metrics can be derived. Network measures that have been found to be altered in focal epilepsy include the efficiency, which is a measure of network integration. It assesses the length and density of connections between regions via both direct and indirect paths (Taylor et al., 2018). Betweenness centrality quantifies the centrality of a brain region within the network. It is defined as the fraction of shortest paths connecting regions A and B that pass through a given region C to the total number of shortest paths connecting A and B. High betweenness values indicate regions that serve as intermediaries for many connections between other regions within the network (Nissen et al., 2016). Cost is calculated as the actual number of edges in the graph relative to the total number of possible edges. Indeed, longer axonal projections are more costly in terms of both material and energy expenditure and space requirements. Average path length refers to the minimum number of edges that must be traversed to travel between any two nodes in the network. This metric is inversely related to the level of network integration (Van Diessen et al., 2014). The clustering coefficient quantifies the number of connections that exist between the nearest neighbours of a node as a proportion of the maximum number of possible connections. The degree is the number of connections that link it to the rest of the network; it is the most fundamental network measure. Most other measures are ultimately linked to node degree (Bullmore and Sporns 2009b).

The aim of this project is to estimate functional network characteristics, which may help predicting outcome of epilepsy surgery, based on the analysis of Resting State directional connectivity using advanced signal processing of non-invasive High Density EEG data. This approach is based on the premise that the epileptic network has abnormal connectivity to neighboring neural structures during the recordings even in the absence of interictal or ictal epileptic discharges (Coito et al., 2019, Nissen et al., 2016, Engel et al., 1993).

## 2 Material and methods

**Fig. 1:**
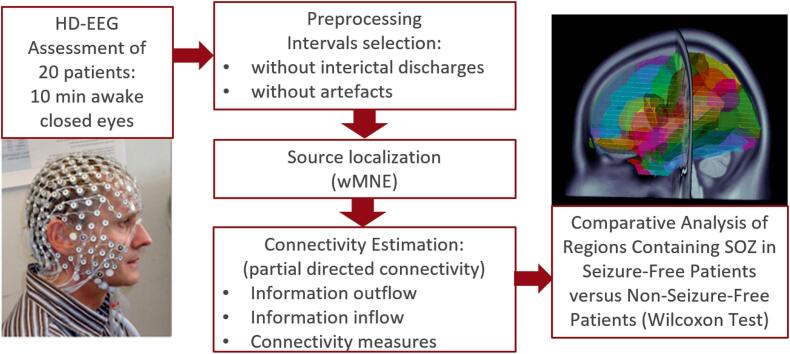
Summary of the analysis strategy.

The analysis strategy is outlined in Fig. 1.

### 2.1 Study cohort

We reviewed consecutive subjects who were admitted to our ward from February 2016 to May 2019. Inclusion criteria were clinical necessity for presurgical work-up with intracranial electrodes, age 17 years and older and subsequent epilepsy surgery. Exclusion criteria were quality deficiency in HD-EEG (one patient was excluded because the recording contained almost continuous artifacts due to eye movements), previous brain surgery, large lesions on MRI, ongoing neurostimulation, progressive disease and mental disability. Patients provided written consent for the in-house use and analysis of their data. Demographic data were derived from patient records. After the presurgical work-up the patients were discussed in an interdisciplinary case conference. According to the recommendation of the case conference 6 patients underwent right temporal lobe resection with amygdalohippocampectomy, 3 patients left temporal lobe resection with amygdalohippocampectomy, 1 patient right temporal lobe resection without amygdalohippocampectomy, 3 patients left temporal lobe resection without amigdalohippocampectomy, 2 patients right selective amygdalohippocampectomy, 1 patients right temporo-lateral resection, 3 patients right frontal resection and 1 patient left frontal resection (Table 1). After the surgery and a follow-up interval of 1 to 7 years patients were classified in seizure-free (Engel 1a according with Engle classification (Engel J, Van Ness PC, Resmussen TB 1993) and non-seizure free (Engel > 1a).

### 2.2 Electroencephalography data acquisition and preprocessing

All patients underwent a HD-EEG (256 channels, Geodescics NA 400 DC amplifier) and were instructed to remain awake with eyes closed during at least 10 min. Impedances were kept as low as possible for all the electrodes. The sampling frequency of the recordings was 1000 Hz. All datasets were filtered offline with a digital high pass filter with a cutoff frequency of 0.5 Hz to remove DC components and slow drifts in the signal. We visually inspected all analyzed signals and topographies and removed bad channels. Cardiac and blinking artefacts were corrected using an independent component analysis (ICA) as implemented in eeglab (Delorme and Makeig 2004). Ten epochs of 5 s duration, free of artifacts and IEDs, were selected per subject.

### 2.3 Source space projection

To accurately model subject-specific anatomy we computed the head models using T1 weighted sequences performed with 3 T scanners (Siemens Magnetom Trio or Prisma) within the framework of presurgical evaluation. A three compartment boundary element model (BEM) was used to solve the EEG forward problem (Gramfort et al. 2010) and Weighted Minimum Norm Estimate (wMNE) implemented in Brainstorm (Tadel et al. 2011) for the estimation of the time courses in the cortical source space. The brain was parceled into 104 regions using the AAL atlas (Tzourio-Mazoyer et al. 2002). These regions were regrouped in 12 regions: frontal right/left, temporomedial right/left, temporolateral right/left, parietal right/left, occipital right/left and thalamus right/left. For each region a representative time course was computed using for each sample the average source strength of the sources of the region.

### 2.4 Electroencephalography network functional connectivity

Patient specific resting state functional connectivity was calculated for each region from 10 epochs, each 5 s long. Directional connectivity between the brain structures was estimated by partial directed coherence (Baccalá and Sameshima 2001). In a first step parameters of a multivariate autoregressive model with a model order of 40 were estimated by the Nutall-Strand algorithm with unbiased covariance (Schlögl 2006). Partial directed coherence is then determined as implemented in the Biosig Toolbox (https://biosig.sourceforge.net) (Schölgl, Vidaurre, and Sander 2011).

Inflow and outflow were estimated for the single anatomical regions in standard EEG frequency bands (Delta: 1.0 Hz – 4.0 Hz, Theta: 5.0 Hz – 8.0 Hz. Alpha: 9.0 Hz – 12.0 Hz, Beta: 13 Hz – 30 Hz).

We investigated local properties of the networks using measures well established in graph theory including local efficiency, betweenness centrality, cost, clustering coefficient, average path length and degree (Bröhl et al., 2023, Bullmore and Sporns, 2009, Carboni et al., 2019, Carboni et al., 2020, Nissen et al., 2016, Taylor et al., 2018, Van Diessen et al., 2014). These were implemented using the functional connectivity toolbox CONN (https://web.conn-toolbox.org/) version 17 (Nieto-Castanon and Whitfield-Gabrieli 2022).

### 2.5 Statistical analysis

To evaluate group-level statistics of the connectivity analyses between seizure free patients and non-seizure free patients after surgery, statistical testing was performed using R V 4.3.0. Our null hypothesis was that inflow, outflow and the other connectivity measures are not different in seizure free versus non-seizure free patients. We tested this hypothesis calculating the significance with Wilcoxon test for independent samples. P-values were not corrected for multiple testing. Boxplots were generated to visualize the results.

### 2.6 Data availability

The data supporting the findings of this study are available upon request from the corresponding author. However, please note that the data are not publicly available due to privacy or ethical restrictions.

## 3 Results

A total of 20 patients (11 females, 9 males, mean age 33 years) met the inclusion criteria and participated in the study. Table 1 displays demographics, clinical data and postoperative outcome (seizure-free vs. non-seizure-free patients). The presence of a lesion in the MRI (magnetic resonance imaging), age at surgery, age at epilepsy onset, duration of epilepsy prior to the first surgery and type of surgery did not differ significantly between patients that were seizure-free and not seizure-free surgery, respectively. MRI revealed hippocampal sclerosis in 4 patients, focal cortical dysplasia in 3 patients, and meningoencephaloceles in 4 patients (2 of them bilateral). In one patient, a meningoencephalocele coexisted with a temporo-occipital meningioma and a cystic lesion in the angular gyrus.

**Table 1:** Demographic and clinical features. *Age at first surgery in those patients who underwent several operations.

Characteristics	Total Patients (n = 20)	Seizure free (n = 10)	Non-seizure free (n = 10)
Age at the surgery* (years) mean/median	33/34	35.43/35.95	31/30.31
Sex (Male/Female)	9/11	6/4	3/7
Age at epilepsy onset (years) mean/median	19/14.5	19.66/16.43	17.69/14.74
Duration of epilepsy prior to first surgery, years			
Mean	15	15.77	13.31
Median	11	10.94	12.26
Range	4–46	4–46	4–33
Lesion visible in MRI, n (%)	11 (52.4)	6 (60)	5 (50)
Type of surgery, n (%)			
right selective amygdalohippocampectomy	2 (10)	2 (20)	0 (0)
right temporal lobe resection with amygdalohippocampectomy	7 (35)	3 (30)	4 (40)
right temporal lobe resection without amygdalohippocampectomy	1 (5)	1 (10)	0 (0)
left temporal lobe resection with amygdalohippocampectomy	3 (15)	3 (30)	0 (0)
left temporal lobe resection without amygdalohippocampectomy	2 (10)	0 (0)	2 (20)
right temporolateral resection	1 (5)	0 (0)	1 (10)
right frontal resection	3 (15)	1 (10)	2 (20)
left frontal resection	1 (5)	0 (0)	1 (10)
Follow-up in the study, months			
Mean	46.65	50.32	42.99
Median	51	51.62	46.50
Range	11–85	12–85	11–70

**Fig. 2:**
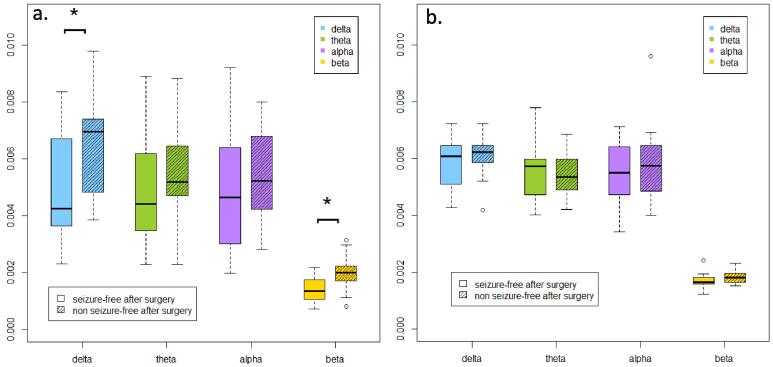
**Boxplots of inflow (a) and outflow (b) in seizure-free patients post-surgery versus those with ongoing seizures.** Inflow was significantly lower in seizure-free patients in the delta (p = 0.026) and beta (p = 0.012) frequency bands (* denotes significant results). In theta and alpha frequency bands, it was lower in seizure-free patients, but results were not significant. Outflow was lower in the delta, alpha, and beta frequency bands, while showing a tendency for higher levels in the theta frequency band among seizure-free patients, also without reaching statistical significance.

To quantify the directional connectivity of the region containing the SOZ with other brain regions, we measured inflow and outflow for individual anatomical regions in standard EEG frequency bands in each participant using pre-surgery HD-EEG. Fig. 2 illustrates inflow and outflow in patients who achieved seizure freedom after surgery compared to those who continued to experience seizures post-surgery. Notably, inflow was significantly lower in seizure-free patients within the delta (p = 0.026) and beta frequency bands (p = 0.012), implying a weaker mean connectivity relative to the patients who didn’t achieve seizure freedom. Although inflow was lower in seizure-free patients within the theta and alpha frequency bands, these differences did not reach statistical significance. Conversely, outflow (Fig. 2b.) was lower across the delta, alpha, and beta frequency bands in seizure free patients, while showing a tendency for increased levels in the theta frequency band among seizure-free patients, also without reaching statistical significance.

**Fig. 3:**
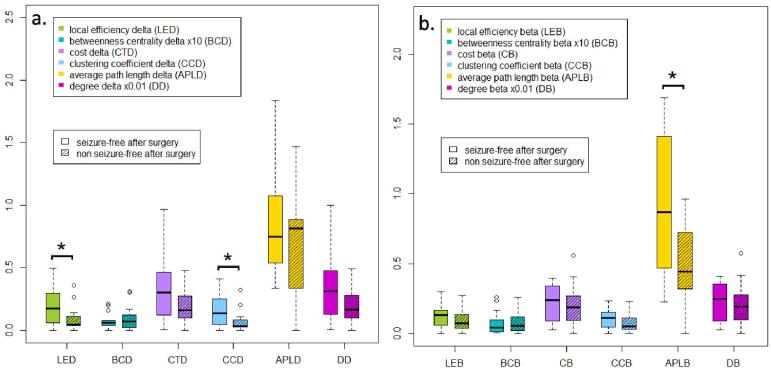
**Boxplots of the graph measures in the delta (a) and beta frequency bands (b), comparing seizure-free patients post-surgery to those with ongoing seizures**. In the delta frequency band, local efficiency (LED) and clustering coefficient (CCD) were significantly higher in seizure-free patients (* denotes significant results). In the beta frequency band, the average path length (APLB) was significantly higher in seizure-free patients, although the distribution of results exhibited considerable variability.

A different intrinsic network pattern was observed in the regions containing the seizure onset zone between seizure-free and non-seizure-free patients after surgery. Local efficiency and clustering coefficient were higher in seizure-free patients, with significant results in the delta frequency band (Fig. 3a). Average path length showed considerable variability in the results but was also higher on the group level in seizure-free patients, with significant differences observed in the beta frequency band (Fig. 3b). While cost and degree tended to be higher in all frequency bands in seizure-free patients, the results were not significant. The distribution of betweenness centrality was similar between seizure-free and non-seizure-free patients after surgery (Supplementary figure 1).

## 4 Discussion

We investigated the association of surgical outcomes after epilepsy surgery and presurgical resting state network brain connectivity. Our objective was to identify EEG resting state pattern that would predict surgical outcome from data acquired prior to surgery. We found distinct connectivity pattern in the delta and beta bands that allow differentiating between seizure free and non-seizure free patients, using only HD-EEG resting state fluctuation in the absence of detectable epileptic discharges.

In patients who achieved seizure freedom after surgery, the preoperative analysis of the epileptic network exhibited stronger separation of the region containing the seizure onset zone with less inflow of information in the delta and beta frequency bands. This result is consistent with the findings of Aydin et al. (Aydin et al. 2020). Using MEG, they identified weaker connections between the brain regions where interictal epileptic discharges are generated and the rest of the cortex in patients who achieved seizure freedom after surgery. However, unlike our study, the differences were observed in the alpha frequency band. Other studies using intracranial EEG and fMRI detected dynamic changes of the effective connectivity in the SOZ of temporal epilepsy patients also in the delta and beta frequency bands (Bettus et al., 2011, Wilke et al., 2011).

Coito et al. found different outflow patterns between right and left temporal lobe epilepsy (Coito et al., 2015) and in the resting state of temporal lobe epilepsy compared with healthy controls (Coito et al. 2016). While the outflow of the epileptic network may indeed be altered, our analysis showed no significant differences when comparing patients who achieved seizure freedom with those who continued to have seizures after surgery. Therefore, we could not corroborate its prognostic value. This should be investigated further with larger patient cohorts.

Analyzing the graph metrics of regions containing the SOZ, we observed higher values for local efficiency and clustering coefficient in the delta frequency band among patients who achieved seizure freedom after surgery. Both metrics describe how much the node is clustered with the neighbors. We conducted the graph metric analysis within each defined region, thus assessing the relationship of the SOZ within the brain lobe rather than on a whole-brain scale. The average path length in the beta frequency band was lower in patients who continued to have seizures after surgery. According to our data, well-structured and clustered epileptic networks are associated with a better surgical prognosis and are more likely to result in seizure freedom after surgery. In contrast, shorter paths within the epileptic network may facilitate hypersynchronous neuronal activity, leading to the recurrence of seizures in patients who do not achieve seizure freedom. The different patterns of connectivity in specific frequency bands (delta for local efficiency and clustering coefficient; beta for average path length) may relate to spatial characteristics of the epileptic network.

Other graph metrics, such as betweenness centrality, cost, and degree, did not demonstrate prognostic value in our study. Wilke et al. found that betweenness centrality correlates with the location of resected regions in patients who were seizure-free following surgical intervention, but they did not compare the results with non-seizure-free patients (Wilke, Worrell, and He 2011). Nissen et al. found high betweenness centrality in the lobe of resection in seizure-free patients compared to non-seizure-free patients (Nissen et al., 2016). A larger cohort of patients may be necessary to reproduce these findings with our analysis approach.

To our knowledge, this is the first study to demonstrate that inflow pattern and graph metrics such as local efficiency, clustering coefficient, and average path length, calculated using non-invasive EEG, could serve as presurgical biomarkers for predicting surgical outcomes. Previous studies have already shown different patterns of connectivity with a loss of distant connections and increased local connections in the vicinity of epileptic focus in temporal lobe epilepsy (Kudo et al., 2022, Liao et al., 2010, Luo et al., 2014, Maneshi et al., 2014, Wilke et al., 2011) and in frontal lobe epilepsy (Luo et al. 2014; Pedersen et al. 2017). However, their prognostic value and relationship with surgical outcomes were not studied.

This study has limitations. First, the small patient cohort limits the generalization of our results. Additionally, the studied patient population is heterogeneous, including 16 temporal lobe epilepsy patients and 4 frontal lobe epilepsy patients. However, it is important to note that we identified connectivity patterns related to seizure freedom even within this heterogeneous group. In our study, as in any functional connectivity analysis, our findings may be influenced by specific decisions regarding the selection of source localization methods and connectivity metrics. We chose wMNE because, in a comparative study including a couple of inverse methods and connectivity estimates, robust results in the majority of combinations of methods and application scenarios were obtained (Hassan et al., 2014).

## 5 Conclusion

We have shown that specific resting-state connectivity patterns, derived from EEG recordings prior to surgery, coupled with precise source localization, can provide important information related to surgical outcome. According to our results, the resection of resting state networks characterized by: 1. weaker connectivity between the region with the SOZ and the rest of the cortex, and 2. a well-connected structure internally, are associated with a seizure free outcome. Conversely, a more extensive epileptic network, marked by enhanced connectivity between the region containing the SOZ and the remainder of the cortex and with a scattered internal structure, serves as a predictor of an unfavorable surgical outcome (Aydin et al. 2020).

## CRediT authorship contribution statement

**Eva Martínez Lizana:** Conceptualization, Data curation, Formal analysis, Investigation, Methodology, Validation, Visualization, Writing – original draft, Writing – review & editing. **Armin Brandt:** Formal analysis, Methodology, Validation, Writing – review & editing. **Matthias Dümpelmann:** Conceptualization, Data curation, Formal analysis, Investigation, Methodology, Validation, Visualization, Writing – review & editing. **Andreas Schulze-Bonhage:** Supervision, Validation, Visualization, Writing – review & editing.

## Declaration of Competing Interest

The authors declare that they have no known competing financial interests or personal relationships that could have appeared to influence the work reported in this paper.

## Data Availability

The data that has been used is confidential.
