# Supplementary figures and images for: Resting state connectivity biomarkers of seizure freedom after epilepsy surgery
Source: Neuroimage Clin. 2024 Sep 16;44:103673. doi: 10.1016/j.nicl.2024.103673 (PMC11424789; doi:10.1016/j.nicl.2024.103673)

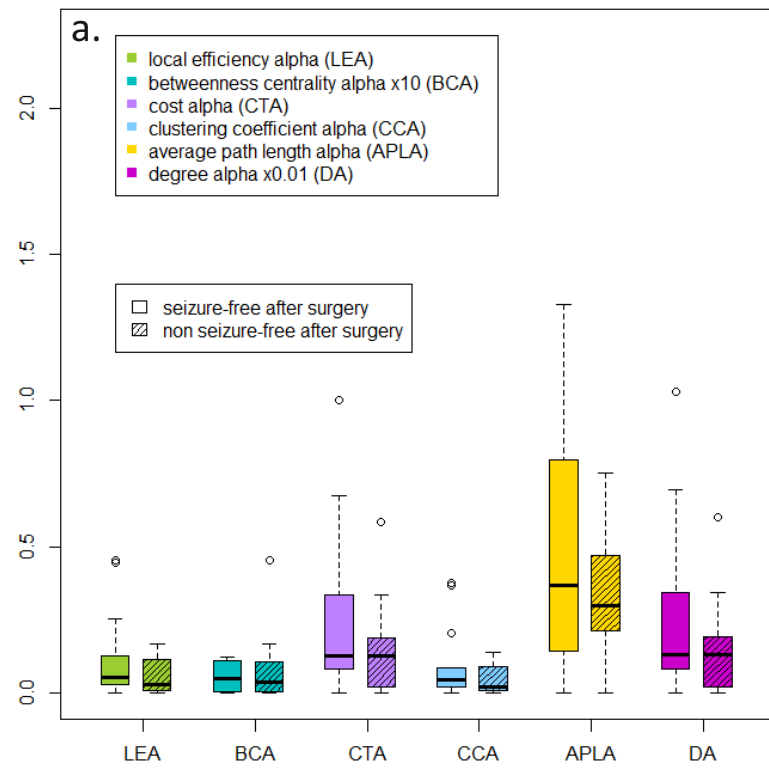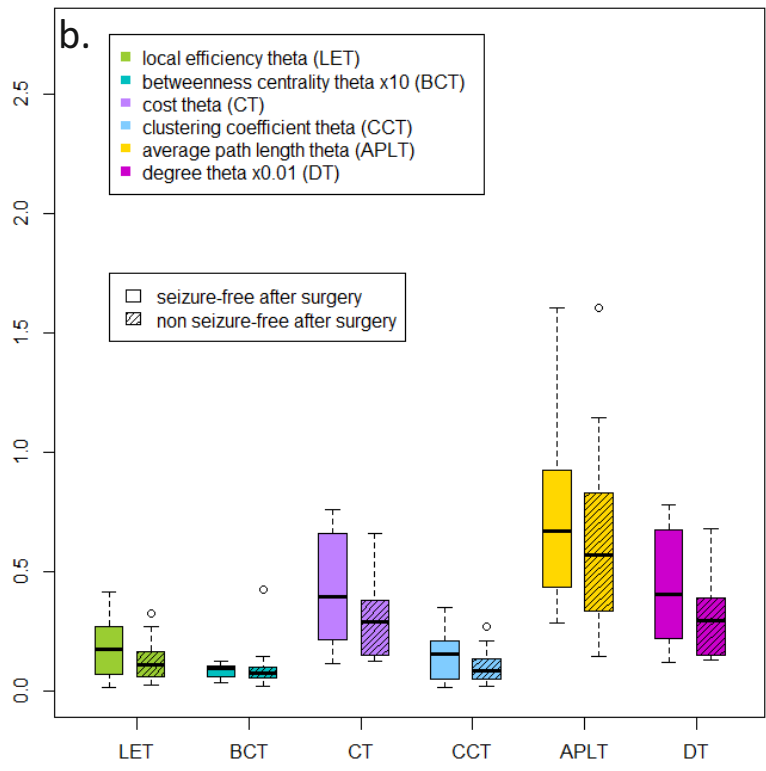

Supplement: Supplementary Data 1 [file mmc1.pdf]
